# Accessibility and use of organised stroke care and reperfusion therapies in Europe: results from the SAP-E Stroke Service Tracker 2023

**DOI:** 10.1093/esj/aakag083

**Published:** 2026-07-21

**Authors:** Lejla Wassvik, Christian Ovesen, Ales Tomek, Hrvoje Budincevic, Diana Aguiar de Sousa, Josefine Grundtvig, Bo Norrving, Francesca Romana Pezzella, Melinda Berg Roaldsen, Simona Sacco, Gustavo Santo, Björn Logi Thórarinsson, Cristina Tiu, Aleksandras Vilionskis, Arlene Wilkie, Hanne Christensen

**Affiliations:** Department of Neurology, Copenhagen University Hospital, Bispebjerg Hospital, Copenhagen, Denmark; Master in Stroke Science Program, University of Bern, Bern, Switzerland; Department of Neurology, Copenhagen University Hospital, Bispebjerg Hospital, Copenhagen, Denmark; Department of Neurology, Motol University Hospital, Prague, Czech Republic; Stroke and Intensive Care Unit, Department of Neurology, Sveti Duh University Hospital, Zagreb, Croatia; Department of Neurology and Neurosurgery, Faculty of Medicine, Josip Juraj Strossmayer University of Osijek, Osijek, Croatia; Stroke Center, Department of Neurosciences, ULS São José, Lisbon, Portugal; Faculdade de Medicina, Universidade de Lisboa, Lisbon, Portugal; Department of Neurology, Copenhagen University Hospital, Bispebjerg Hospital, Copenhagen, Denmark; Department of Clinical Sciences, Lund University, Lund, Sweden; Department of Neurosciences, Azienda Ospedaliera San Camillo Forlanini, Rome, Italy; Department of Clinical Medicine, UiT The Arctic University of Norway, Tromsø, Norway; Centre of Digitalization, Development and Integrative Care, University Hospital of North Norway, Tromsø, Norway; Department of Biotechnological and Applied Clinical Sciences, University of L'Aquila, L'Aquila, Italy; Department of Neurology, University Hospital of Coimbra, Unidade Local de Saúde de Coimbra, Coimbra, Portugal; Department of Neurology, Landspitali University Hospital, Reykjavik, Iceland; Department of Neurology, University Hospital Bucharest, Bucharest, Romania; Department of Clinical Neurosciences, Carol Davila University of Medicine and Pharmacy, Bucharest, Romania; Department of Neurology and Neurosurgery, Faculty of Medicine, Vilnius University, Vilnius, Lithuania; Stroke Alliance for Europe, Brussels, Belgium; Department of Neurology, Copenhagen University Hospital, Bispebjerg Hospital, Copenhagen, Denmark

**Keywords:** ESO, Stroke Action Plan for Europe (SAP-E), Stroke Alliance for Europe, stroke treatment, healthcare resources, stroke unit, intravenous thrombolysis, endovascular treatment

## Abstract

**Introduction:**

Organised stroke care and reperfusion therapies are key components of modern stroke treatment. We assessed accessibility of organised stroke care and reperfusion therapies in Europe and explored its association with key organisational indicators.

**Patients and methods:**

Stroke Action Plan for Europe (SAP-E) Stroke Service Tracker data from 2023 reported by 47 European countries was assessed. Accessibility indicators included stroke unit (SU) admission proportions, intravenous thrombolysis (IVT) and EVT treatment proportions. Organisational indicators included the facility density of SU, IVT-capable centres and EVT-capable centres per capita (number of centres per 100,000 population), presence of national stroke plans and quality programmes.

**Results:**

Across Europe, marked variations were observed in facility density of SU (0.21–0.48), IVT-capable centres (0.19–0.45) and EVT-capable centres (0.06–0.15). SU admission proportions ranked from 0.8% to 96.7%, IVT treatment proportions from 1.3% to 34.7% and EVT treatment proportions from 0.2% to 14.3%. Stroke unit admission proportions were associated with SU density (*r* = 0.42, *P* = .03). Density of reperfusion-capable centres showed a possible trending association with IVT treatment proportions (*r* = 0.34, *P* = .08), but no association with EVT treatment proportions (*r* = 0.22, *P* = .23). Neither the presence of a national stroke plan nor a quality programme were associated with accessibility of SU care, IVT or EVT.

**Conclusion:**

Large inequities in access to organised stroke care and reperfusion therapies persist across Europe. At the country level, national stroke plans and quality programmes have not yet translated into effect on accessibility indicators, suggesting that achieving effective implementation takes time or could primarily manifest as within-country improvements.

## Graphical Abstract

**Graphical Abstract:**
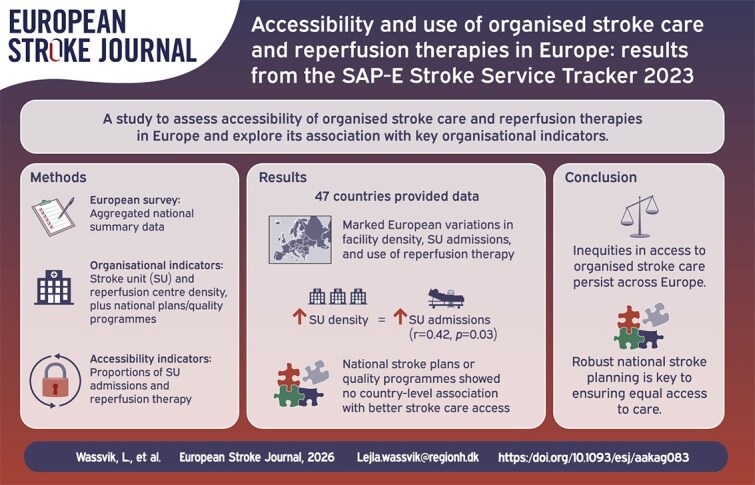

## Introduction

Organised stroke unit (SU) care, intravenous thrombolysis (IVT) and EVT are the cornerstones of acute stroke management.^1–5^ Evidence demonstrates that each intervention independently enhances clinical outcomes, with time-dependent benefits that increase when delivered promptly.^6–8^ Despite these benefits, access to SU care and reperfusion therapies varies substantially across Europe,^2^^,^^3^^,^^9–11^ which likely contributes to stroke being one of the leading causes of mortality and disability worldwide.^9^^,^^12^ Differences in stroke service organisation, distribution of specialised centres and geographic factors are likely key contributors to persistent disparities.^13^

To address these challenges, the Stroke Action Plan for Europe (SAP-E) 2018–2030 was developed jointly by the ESO and the Stroke Alliance for Europe.^13–16^ Stroke Action Plan for Europe aims to improve stroke prevention, acute care, rehabilitation and long-term support across Europe. To facilitate implementation, SAP-E has defined 13 key performance indicators (KPIs), including the presence of national stroke plans and quality programmes, rates of hospital admission to SU, utilisation of IVT and EVT and process times such as door-to-needle (DTN) and door-to-groyne (DTG) times.^17^ These indicators are annually monitored through the SAP-E Stroke Service Tracker (SST). The most recent SST data indicate that substantial inequities in access to organised stroke care and reperfusion therapies are still present across Europe.^13^

The aim of this analysis is to map the organisation and accessibility of organised stroke care across Europe, and to evaluate the relationship between the organisation and the accessibility of stroke care.

## Methods

### Stroke Service Tracker

This cross-sectional observational study included the data reported for the year 2023 in SST from all 47 participating countries.^13^ The methodology of the SST database as well as results from the 2023 dataset (including SU admission rates, rates of IVT and EVT treatment as well as DTN and DGT times, which are also presented in this manuscript) have previously been published.^11^ In brief, aggregate summary data and KPIs from all European countries are collected through an annual survey. National coordinators respond to the survey based on the best available data. All national coordinators are leading stroke experts. Data were classified as “high-quality data,” when information was derived from national or regional stroke registries, reimbursement databases, or national surveillance systems. “Lower quality data” included expert estimates, institutional registries and direct contact with sites. As this study was based on country-level aggregate data and did not include individual patient-level information, ethical approval and informed consent were not required. Data are uploaded and stored in a database hosted by the Capital Region of Denmark. Only the project leadership can access the entire database. Entries in the SST database are monitored by 2 stroke experts from the SAP-E organisation. Any data queries are sent to the national coordinators, and data are only accepted as complete after adequate responses.

### Definition of accessibility of stroke care and organisation indicators

In this study, accessibility of acute stroke care was defined as the ability of individuals to obtain timely, evidence-based diagnostic and treatment services. Accessibility of organised stroke care and reperfusion therapy was pragmatically estimated using 5 key indicators: the proportion of stroke patients receiving SU admission (including both ischemic and haemorrhagic stroke), IVT treatment proportions and EVT treatment proportions as well as DTN and DTG times. This approach acknowledges that “accessibility” is a complex concept involving both geographical and systemic elements that is difficult to capture pragmatically. The SU admission proportion represents the proportion of stroke patients (including both ischemic and haemorrhagic stroke patients) receiving SU care. It was chosen to include both subtypes in the SU admission proportion, as SU care is core evidence-based treatment for both subtypes. However, IVT treatment proportions and EVT treatment proportions only represent the proportion of ischemic stroke patients undergoing the respective treatment.

Organisational indicators included the density (national number of units per 100,000 population) of SU, SU beds, IVT-capable centres and EVT-capable centres. A SU is defined as a dedicated hospital ward providing organised and multidisciplinary care for stroke patients with a focus on acute treatment, complication prevention and early rehabilitation. Additional key organisational indicators included the implementation status of a national stroke plan and the presence of a national quality monitoring programme (defined by existing national registries, national clinical guidelines, audits or accreditation programmes).^13^ Countries were geographically grouped according to the United Nations geoscheme into Northern, Western, Eastern and Southern Europe, as well as Western Asia.^18^

### Statistical analysis

The presented data are summary data. Descriptive data are reported as counts (percentage) or medians (IQR) as appropriate. The association between key categorical variables (eg, implementation status of a national stroke plan or geographical region) and continuous indicators (eg, rate of stroke unit admission or intravenous thrombolysis rate) was visualised using box plots (displaying median and IQR) and compared using the Kruskal–Wallis test. The Pearson correlation coefficient was used to assess the strength of the linear association between continuous indicators and continuous organisational parameters (eg, density of stroke units). The hypothesis that the correlation coefficients differed from zero was non-parametrically tested through 10,000 permutation re-samples of the dataset. All analyses were conducted using Stata 18.0 (StataCorp, TX, USA).

## Results

### Organisation of stroke care

Organisation of SU care and reperfusion therapy is presented in Table S1. Among the 38 reporting countries, the median (IQR) SU density was 0.39 (0.21–0.48) per 100,000 population, ranging from 0.06 in Kosovo to 0.86 in Norway. For the 25 countries reporting on SU beds, the median (IQR) density was 3.1 (1.3–6.2) per 100,000 population, ranging from 0.06 in Greece to 30.0 in the Czech Republic. Among the 36 countries reporting on the availability of IVT-capable centres, the median (IQR) density was 0.38 (0.19–0.45) per 100,000 population, ranging from 0.06 in Kosovo to 0.86 in Norway. The median (IQR) density of EVT-capable centres (reported by 38 countries) was 0.10 (0.06–0.15) per 100,000 population, ranging from 0.03 in Wales to 0.37 in Finland. National stroke plans were present in 20 (43.5%) of the 47 participating countries, 19 (41.3%) reported that work on a national stroke plan was ongoing and 7 (15.2%) reported having no national stroke plan. In addition, 20 (43.5%) countries reported having an established national quality programme (Table S1).

### Organisation of stroke care and accessibility of stroke care

An overview of the accessibility of SU care and reperfusion therapies in each country, alongside data quality assessments, is presented in Table S2 and Figure S1. Marked disparities were observed across all 5 accessibility parameters: SU admission, IVT and EVT proportions and DTN and DTG times (Figures S2 and S3).

**Figure 1:**
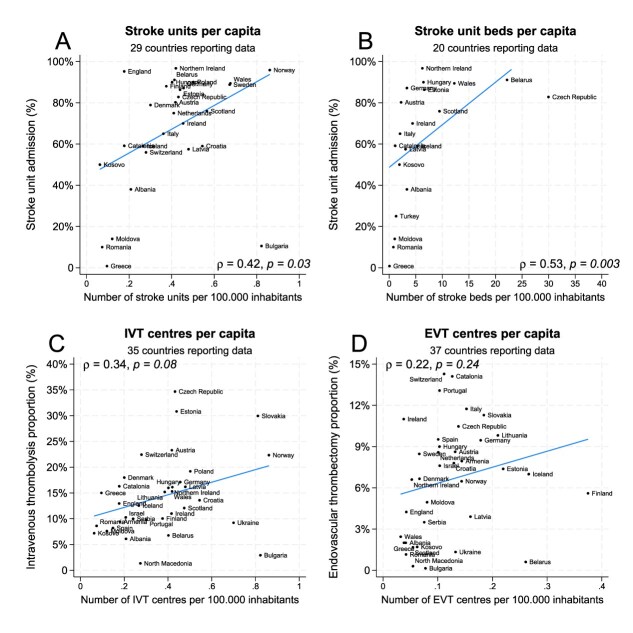
Association between facility density and accessibility of stroke care. Panels A and B show the association between the density of stroke units and stroke unit beds (no. per 100,000 population) and the proportion of stroke unit admissions. Panels C and D illustrate the association between the density of centres capable of providing reperfusion therapy (per 100,000 population) and the proportions of patients receiving such treatment. Abbreviation: IVT = intravenous thrombolysis.

Among all reporting countries, SU admission proportions were positively associated with SU density (*r* = 0.42, *P* = .03; Figure 1) and the density of SU beds (*r* = 0.53, *P* = .003). A possible weak association was observed between IVT treatment proportions and the density of IVT-capable centres (*r* = 0.34, *P* = .08), whereas no such association was observed between EVT treatment proportions and the density of EVT-capable centres (*r* = 0.22, *P* = .24). Regarding treatment delay, an association was found between the density of EVT-capable centres and DTG time (*r* = −0.39, *P* = .04; Figure S4); however, there was uncertainty regarding the association between the density of IVT-capable centres and DTN time (*r* = −0.36, *P* = .08). While a possible weak association was observed between IVT treatment proportions and the SU density (*r* = 0.32, *P* = .08; Figure S5), no such association was shown between EVT treatment proportions and the SU density (*r* = 0.01, *P* = .96). When restricting to countries providing “high-quality data,” the association between SU admission proportions and the SU density has attenuated (*r* = 0.45, *P* = .09; Figure S6). For the remaining estimates, no significant associations were observed among countries providing “high-quality data” (Figures S6–S8).

**Figure 2:**
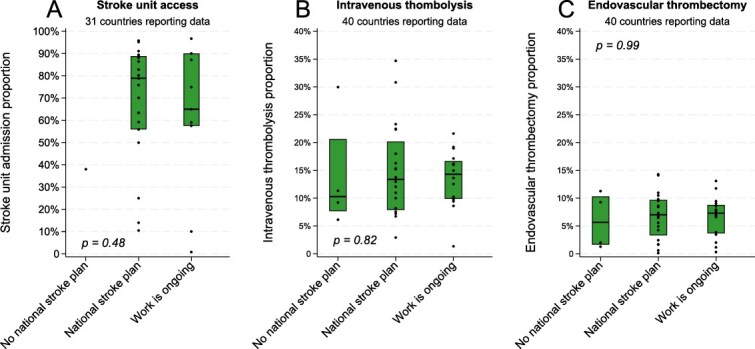
Accessibility of stroke care stratified by national stroke plan implementation status. Bars represent the median (interquartile range) proportions of stroke unit admissions (panel A), intravenous thrombolysis (panel B) and endovascular thrombectomy (panel C). Individual country-level data points are superimposed as dots.

**Figure 3:**
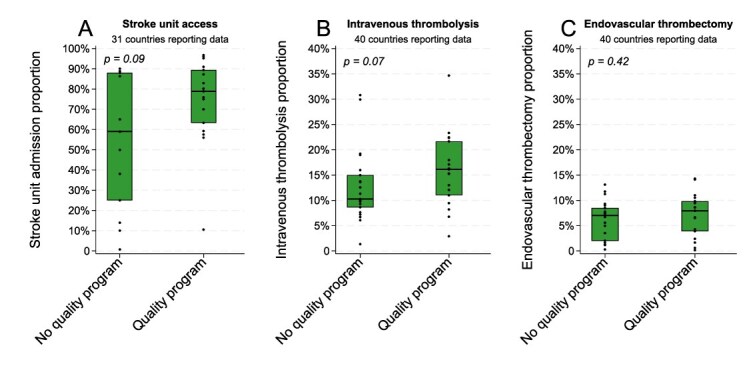
Accessibility of stroke care stratified by the presence of a quality programme. Bars represent the median (interquartile range) proportions of stroke unit admissions (panel A), intravenous thrombolysis (panel B) and endovascular thrombectomy (panel C). Individual country-level data points are superimposed as dots.

National stroke plan implementation status was not found to be associated with differences in SU admission (*P* = .48; Figure 2), IVT (*P* = .82) or EVT proportions (*P* = .99). Similarly, no differences were observed in DTN (*P* = .20; Figure S9) or DTG (*P* = .20) times when comparing countries by national stroke plan status. In contrast, countries with an established quality programme showed a marginal trend towards higher median [IQR] SU admission proportions (78.9% [63.3%–89.4%] vs 59.0% [25.0%–88.1%], *P* = .09; Figure 3), higher IVT treatment proportions (16.1% [11.0%–21.7%] vs 10.3% [8.6%–15.0%], *P* = .07), as well as shorter DTN times (Figure S10). No differences were observed in EVT treatment proportions (*P* = .42; Figure S3) or DTG times (*P* = .24; Figure S10) according to quality programme status. When restricting to countries providing “high-quality data,” no differences were observed in any of the estimates according to national stroke plan implementation status or quality programme status (Figures S11–SS14).

### Geographic regions and accessibility of stroke care

**Figure 4:**
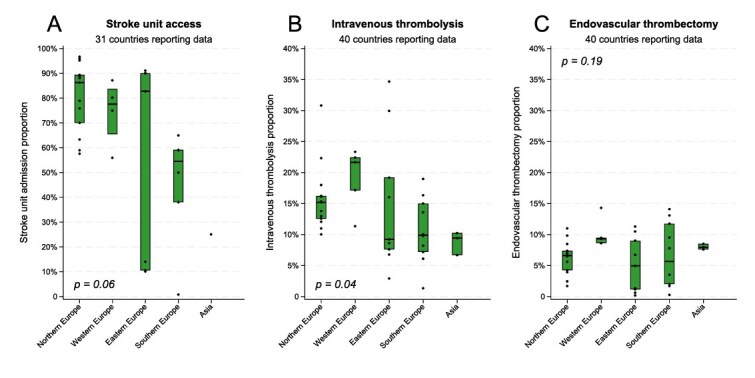
Accessibility of stroke care stratified by geographic regions (UN geoscheme regions). Bars represent the median (interquartile range) proportions of stroke unit admissions (panel A), intravenous thrombolysis (panel B) and endovascular thrombectomy (panel C). Individual country-level data points are superimposed as dots.

Stroke unit admission proportions showed only marginal variation by geographic region (*P* = .06; Figure 4). Median [IQR] admission proportions were higher in Northern (86.3% [70.0%–89.4%]), Western (77.6% [65.5%–83.7%]) and Eastern Europe (82.2% [10.6%–90.0%]) compared to Southern Europe (54.5% [38.0%–59.1%]). When comparing geographic regions, IVT treatment proportions showed weak regional variation (*P* = .04; Figure 4). Northern (15.2% [12.6%–16.2%]) and Western Europe (21.7% [17.1%–22.4%]) reported higher median (IQR) proportions as compared to Eastern Europe (9.2% [7.6%–19.2%]), Southern Europe (9.9% [7.2%–15.0%]) and Western Asia (9.4% [6.7%–10.3%]). In contrast, EVT treatment proportions (*P* = .19; Figure 4) as well as DTN or DTG times (Figure S15) did not differ across geographic regions. When restricting to countries providing “high-quality data,” an association was observed between EVT treatment proportion and geographic regions (*P* = .02; Figure S16), characterised by higher reported proportions in southern Europe. No other estimates showed regional variation among “high-quality data” countries (Figures S16 and S17).

## Discussion

In this analysis of 2023 SAP-E SST data, access to organised SU care and reperfusion therapy varied substantially across Europe. Across reporting countries, facility density was associated with SU accessibility and showed a weak potential trend with IVT treatment proportions, whereas no association was observed for EVT proportions. Treatment delay was associated with EVT-centre density (DTG time), but not with IVT-centre density (DTN time). No clear associations were observed between the accessibility indicators and either national stroke plan status or the presence of a national quality programme. Finally, data indicated potential variations in stroke care accessibility among geographic regions.

Overall, these findings highlight inequities in evidence-based acute stroke care and variation in national stroke service organisation and resources. This likely reflects different organisational models across Europe. Structural indicators alone are unlikely to capture all determinants of access and quality, as they do not account for prehospital delays, geographic barriers or treatment eligibility and should therefore be interpreted as a proxy of system performance.

Stroke unit admission proportions were associated with SU density; however, countries with high admission proportions clustered within a relatively narrow SU-density range, suggesting that SU density alone does not fully explain access beyond a certain level (particularly in low-population-density or geographically challenging settings).^19^ This pattern is consistent with the importance of additional factors, including admission criteria, catchment areas and geography.^20^ Stroke unit admission proportions were also associated with SU-bed density, yet similar bed capacity coincided with widely different admission proportions. This discordance may reflect differences in beds per SU, transport times, prehospital triage, admission practices and length of stay. Although a possible association was observed between the density of IVT-capable centres and IVT treatment proportions across Europe, similar densities of IVT-capable centres were accompanied by varying IVT treatment proportions between countries. This indicates that IVT utilisation is also influenced by organisational and process-related factors such as prehospital recognition, in-hospital workflows, admission policies, decision-making and out-of-hours service organisation.^21^ In contrast, EVT treatment proportions were not associated with density of EVT-capable centres. Endovascular thrombectomy-capable centres were fewer and more unevenly distributed than SU or IVT-capable centres, consistent with specialised, centralised service models. While centralisation may be necessary to maintain procedural expertise, it poses challenges for geographic access and prehospital triage. Consequently, density of EVT-capable centres alone may not fully account for variations in EVT utilisation; instead, disparities in EVT proportions and DTG times may reflect prehospital identification of large-vessel occlusions, in-hospital workflows, patient selection and the availability of neurointerventionalists.^22^

Implementation status of a national stroke plan and the presence of a national quality programme were not associated with the accessibility indicators, despite a weak trend towards higher SU admission proportions, higher IVT proportions and shorter DTN times in countries with quality programmes. These findings do not diminish the potential importance of strategic planning and quality monitoring; rather, they indicate that the mere presence of a national stroke plan or a quality programme do not ensure equitable access. Meaningful effects likely depend on comprehensive implementation into daily clinical workflows and may take time to become measurable.^13^^,^^23^ In addition, this cross-sectional ecological analysis based on aggregate country-level data cannot assess potential within-country quality improvements resulting from the implementation of a national stroke plan or quality programme. Furthermore, it cannot capture the between-country heterogeneity in programme content or implementation strategies. These limitations preclude inferences about the programmes’ within-country effectiveness.

Analyses of geographic regions revealed possible variations in access to SU care and reperfusion therapies; however, interpretation is limited due to heterogeneity within regions and incomplete data. Access to SU care appeared numerically higher in Northern, Western and Eastern Europe than in Southern Europe, where high-quality data were sparse. Wide within-region variation—particularly in Eastern Europe—suggests that regional groupings may mask country-level differences. The attenuation of several associations in analyses restricted to countries providing “high-quality data” may reflect reduced measurement variability, as these countries often reported data clustering within a similarly performance range compared with analyses incorporating expert estimates and heterogeneous data sources. In addition, restricting the analyses to countries with high-quality data reduced the sample size, which may have limited the statistical power to detect potential associations.

This study has limitations that should be considered when interpreting the findings. First, the analysis is cross-sectional and based on country-level data from SAP-E SST for 2023. As such, all analyses rely on national averages and can only draw inferences at the country-level. Country-level observations are potentially subject to ecological bias and consequently cannot be extended to the individual patient-level or be used to draw causal inferences. However, SAP-E SST represents the only established system for annual surveillance of stroke service organisation and performance across Europe. Second, data completeness and quality varied among SAP-E indicators, and the main analyses relied on data reported by national coordinators using the best available national sources. The descriptive nature of the analyses, combined with the use of country-level variables, precluded meaningful multivariable modelling and left the reported associations potentially susceptible to residual confounding. Consequently, these findings should be considered exploratory in nature. Finally, although the regional grouping based on the UN geoscheme provides an objective framework, its broad regional divisions are somewhat crude and can potentially mask significant regional variations between countries.

## Conclusions

Substantial inequities in access to evidence-based organised stroke care persist across Europe. Organisational capacity, including SU and reperfusion-capable centre availability, was not consistently associated with accessibility indicators, suggesting that structural resources alone do not fully account for disparities in access to evidence-based stroke care. Because this study was not designed to determine causal or explanatory pathways, these findings remain exploratory. At the country level, the presence of national stroke plans and quality programmes showed no clear associations with key accessibility indicators. This may indicate that any impact of such programmes depends on effective implementation in routine clinical practice and may take time to become measurable. However, these findings do not exclude substantial within-country improvements in stroke care associated with such initiatives. Continued efforts should focus on improving stroke care systems and reducing disparities in access across countries.

## Supplementary Material

Supplementary_material_v_1_7_11_5_26_-_1_(3)_aakag083
