# Supplementary material for: Accessibility and use of organised stroke care and reperfusion therapies in Europe: results from the SAP-E Stroke Service Tracker 2023
Source: Eur Stroke J. 2026 Jul 21;11(7):aakag083. doi: 10.1093/esj/aakag083 (PMC13387431; doi:10.1093/esj/aakag083)
Supplement: Supplementary_material_v_1_7_11_5_26_-_1_(3)_aakag083 [file supplementary_material_v_1_7_11_5_26_-_1_(3)_aakag083.pdf]

ACKNOWLEDGEMENTS – COMPLETE LIST OF NATIONAL COORDINATORS:

Albania: Mentor Petrela; Armenia: Marine Balasanyan and Nune Yeghiazaryan; Austria: Stefan Kiechl and Wilfried Lang; Azerbaijan: Rahim Aliyev and Nara Hesen; Belarus: Sergey Marchenko, Yaroslav Zholnerkevich and Tanya Pavlovskaya; Belgium: Sylvie DeRaedt, François Delvoye, and Laetitia Yperzeele; Bosnia and Herzegovina: Renata Jurina, Nevena Mahmutbegović and Marija Bender; Bulgaria: Dorina Dobreva, Dimitar Maslarov and Timitar Taskov; Catalonia: Pere Cardona and Esther Duarte; Croatia: Hrvoje Budincevic, Ervin Jančić and Maša Ožegović; Cyprus: Marian Charalambous, Andreas Charidimou and Georgios Kaponides; Czechia: Magdalena Pohnanová and Aleš Tomek; Denmark: Dorte Damgaard, Birgitte Hysse Forchhammer and Troels Wienecke; England: Sarah Belson, Jatt Khaira and Joseph Kwan; Estonia: Janika Kõrv and Riina Vibo; Finland: Pauli Ylikotila and Susanna Roine; France: Guillaume Turc, Charlotte Cordonnier and Jean Bouchard; Georgia: Alexander Tsiskaridze and Nina Lobajanidze; Germany: Jürgen Faiss, Markus Wagner, Tobias Neumann-Haefelin and Christina Franzisket; Greece: Haralampos Milionis and Hariklia Proios; Hungary: András Folyovich and Tamás Jarecsny; Iceland: Marianne Klinke, Thorir Steingrímsson and Björn Thorarinsson; Republic of Ireland: Rónan Collins and Ciara Breen; Israel: Rani Barnea, Natan Bornstein and Pnina Rosenzweig; Italy: Massimo Del Sette, Fabrizio Pennaccehi, Danilo Toni, Paola Santalucia, Paolo Candelaresi and Ettore Nicolini; Kazakhstan: Zauresh Akhmetzhanova, Sabina Medukhanova and Adilbekov Yerzhan; Kosovo: Dren Boshnjaku and Fisnik Jashari; Kyrgyzstan: Kunduz Karbozova, Asel Kerimkulova and Dzhamalbek Turgumbaev; Latvia: Guntis Karelis and Evija Miglane; Lithuania: Dalius Jatuzis and Aleksandras Vilionskis; Malta: Maria Mallia and Maria Bonello; Moldova: Natalia Ciobanu, Stanislav Groppa and Daniela Efremov; Montenegro: Milovan Roganovic and Sandra Vujovic; Netherlands: Heleen den Hertog and Bert Vrijhoef; North Macedonia: Anita Arsovska, Maja Bozinovska Smicheska, Gordana Dimeska and Elena Lichkova; Northern Ireland: Fiona Quigg and Malcolm Wiggam; Norway: Annette Fromm and Agnethe Eltoft; Poland: Michal Karlinski and Adam Kobayashi; Portugal: Diana Aguiar de Sousa, Elsa Azevedo, Ana Catarina Fonseca, Ana Nunes, Gustavo Santo and Diana Wong

Ramos; Romania: Elena Terecoasa and Cristina Tiu; Scotland: Mary Joan MacLeod, Pamela Maclean and Matthew Lambert; Serbia: Ivan Milojevic, Željko Živanović and Nikola Vukašinović; Slovakia: Zuzana Gdovinova, Vladimir Nosal and Peter Turčáni; Slovenia: Bojana Zvan; Spain: Maria del Mar Freijo and Elena Lopez-Cancio; Sweden: Mia von Euler and Signild Åsberg; Switzerland: Timo Kahles and Krassen Nedeltchev; Turkey: Attila Özcan Özdemir and Mehmet Topcuoglu; Ukraine: Yuriy Flomin, Marina Gulyayeva and Dmytro Lebedynets; Wales: Christine Owen.

SUPPLEMENTARY TABLES

Supplementary Table 1: Overview of the Organisation of Stroke Care

Country	National stroke plan	Quality program	Stroke unit organisation		Organisation of reperfusion therapy	
			Stroke units per 100.000 population (absolute number)	No. stroke unit beds per 100.000 population (absolute number)	IVT centres per 100.000 population (absolute number)	EVT centres per 100.000 population (absolute number)
**Northern Europe**						
Denmark	Yes	Yes	0.30 (18)	NR	0.20 (12)	0.07 (4)
England	Yes	Yes	0.18 (100)	NR	0.18 (100)	0.04 (24)
Estonia	Yes	No	0.44 (6)	6.59 (90)	0.44 (6)	0.22 (3)
Finland	Yes	No	0.37 (21)	NR	0.37 (21)	0.37 (21)
Iceland	Work ongoing	No	0.27 (1)	5.33 (20)	0.27 (1)	0.27 (1)
Latvia	Work ongoing	Yes	0.48 (9)	3.09 (58)	0.48 (9)	0.16 (3)
Lithuania	Yes	Yes	NR	NR	0.38 (11)	0.21 (6)
Northern Ireland	Work ongoing	Yes	0.42 (8)	6.25 (119)	0.42 (8)	0.05 (1)
Norway	Yes	Yes	0.86 (48)	NR	0.86 (48)	0.14 (8)
Republic of Ireland	Yes	Yes	0.45 (24)	4.39 (232)	0.42 (22)	0.04 (2)
Scotland	Yes	Yes	0.57 (31)	9.45 (518)	0.47 (26)	0.05 (3)
Sweden	Yes	No	0.67 (71)	NR	NR	0.07 (7)
Wales	Yes	Yes	0.68 (21)	12.24 (380)	0.42 (13)	0.03 (1)
**Western Europe**						
Austria	Yes	Yes	0.42 (38)	2.25 (205)	0.42 (38)	0.13 (12)
Belgium	No	No	NR	NR	NR	NR
France	Work ongoing	Yes	NR	NR	NR	NR
Germany	Work ongoing	Yes	0.46 (383)	3.33 (2800)	0.46 (383)	0.18 (150)
Switzerland	Yes	Yes	0.28 (25)	NR	0.28 (25)	0.11 (10)
The Netherlands	Work ongoing	Yes	0.41 (73)	NR	NR	0.10 (18)
**Eastern Europe**						
Belarus	Yes	Yes	0.41 (38)	22.18 (2041)	0.40 (37)	0.26 (24)
Bulgaria	Yes	Yes	0.82 (53)	NR	0.82 (53)	0.08 (5)

Czech Republic	Yes	Yes	0.43 (47)	29.97 (3261)	0.43 (47)	0.14 (15)
Hungary	Work ongoing	No	0.40 (39)	6.45 (626)	0.40 (39)	0.10 (10)
Poland	Work ongoing	No	0.50 (189)	NR	0.50 (189)	NR
Republic of Moldova	Yes	No	0.12 (3)	1.05 (26)	0.12 (3)	0.08 (2)
Romania	Work ongoing	No	0.07 (14)	0.79 (150)	0.07 (14)	0.04 (8)
Slovakia	No	No	0.81 (44)	NR	0.81 (44)	0.18 (10)
Ukraine	No	No	0.63 (239)	NR	0.70 (264)	0.13 (50)
**Southern Europe**						
Albania	No	No	0.21 (5)	3.33 (80)	0.21 (5)	0.04 (1)
Bosnia and Herzegovina	No	No	NR	NR	NR	NR
Catalonia	Yes	Yes	0.18 (14)	1.11 (88)	0.18 (14)	0.13 (10)
Croatia	Work ongoing	No	0.54 (21)	NR	0.54 (21)	0.13 (5)
Greece	Work ongoing	No	0.10 (10)	0.06 (6)	0.10 (10)	0.04 (4)
Italy	Work ongoing	No	0.36 (213)	2.05 (1209)	NR	0.15 (90)
Kosovo	Yes	No	0.06 (1)	1.94 (31)	0.06 (1)	0.06 (1)
Malta	Work ongoing	No	0.18 (1)	4.52 (25)	0.18 (1)	0.18 (1)
Montenegro	No	No	NR	NR	NR	NR
North Macedonia	Work ongoing	No	0.27 (5)	NR	0.27 (5)	0.05 (1)
Portugal	Work ongoing	No	0.31 (33)	3.08 (328)	0.30 (32)	0.10 (11)
Serbia	Work ongoing	No	0.24 (16)	NR	0.24 (16)	0.08 (5)
Spain	Yes	Yes	0.15 (72)	0.71 (345)	0.15 (72)	0.10 (49)
**Asia**						
Armenia	Work ongoing	Yes	0.18 (5)	1.51 (42)	0.18 (5)	0.14 (4)
Azerbaijan	No	No	NR	NR	NR	NR
Cyprus	Work ongoing	No	NR	NR	NR	NR
Georgia	NR	NR	NR	NR	NR	NR
Israel	Work ongoing	No	0.21 (20)	1.04 (100)	0.21 (20)	0.10 (10)
Turkey	Yes	No	NR	1.30 (1136)	NR	NR

NR – not reported

Supplementary Table 2: Performance related to stroke unit admission and reperfusion therapy

Country	Stroke unit admission proportion (%)	IVT treatment		EVT treatment	
		IVT treatment proportion (%)	DTN time (minutes)	EVT treatment proportion (%)	DTG time (minutes)
Northern Europe					
Denmark	78.9 [High]	18.0 [High]	28 [High]	6.7 [High]	28 [High]
England	95.2 [High]	13.0 [High]	53 [High]	4.2 [High]	194 [High]
Estonia	86.3 [High]	30.8 [High]	34 [Lower]	7.4 [High]	69 [Lower]
Finland	88.1 [Lower]	10.0 [Lower]	28 [Lower]	5.6 [Lower]	52 [Lower]
Iceland	59.0 [High]	12.6 [High]	35 [High]	7.0 [High]	93 [High]
Latvia	57.5 [Lower]	16.2 [High]	30 [Lower]	3.9 [Lower]	70 [Lower]
Lithuania	63.3 [High]	15.2 [Lower]	31 [Lower]	9.8 [Lower]	95 [Lower]
Northern Ireland	96.7 [High]	16.1 [High]	46 [High]	6.6 [High]	45 [High]
Norway	95.9 [High]	22.3 [High]	30 [High]	6.5 [High]	78 [High]
Republic of Ireland	70.0 [Lower]	11.0 [Lower]	53 [High]	11.0 [Lower]	NR
Scotland	75.9 [High]	12.1 [High]	53 [High]	1.7 [High]	NR
Sweden	88.8 [High]	13.8 [High]	34 [High]	8.5 [High]	122 [High]
Wales	89.4 [High]	15.3 [High]	75 [High]	2.4 [High]	290 [High]
Western Europe					
Austria	80.2 [High]	23.3 [High]	45 [High]	8.6 [High]	86 [High]
Belgium	NR	11.3 [High]	NR	9.3 [High]	NR
France	NR	NR	NR	NR	NR
Germany	87.2 [High]	17.1 [High]	38 [Lower]	9.5 [High]	74 [Lower]
Switzerland	56.0 [High]	22.4 [High]	40 [High]	14.3 [High]	76 [High]
The Netherlands	75.0 [Lower]	21.6 [High]	27 [High]	8.6 [High]	48 [High]
Eastern Europe					
Belarus	91.1 [Lower]	6.8 [Lower]	NR	0.6 [Lower]	NR
Bulgaria	10.6 [Lower]	2.9 [High]	40 [Lower]	0.2 [High]	90 [Lower]
Czech Republic	82.8 [High]	34.7 [High]	20 [Lower]	10.5 [High]	55 [Lower]

Hungary	90.0 [Lower]	16.0 [Lower]	59 [Lower]	9.0 [Lower]	110 [Lower]
Poland	90.0 [High]	19.2 [High]	60 [High]	6.7 [High]	147 [High]
Republic of Moldova	14.0 [Lower]	7.6 [Lower]	59 [Lower]	4.9 [Lower]	118 [Lower]
Romania	10.0 [Lower]	8.6 [Lower]	55 [Lower]	1.1 [Lower]	96 [Lower]
Slovakia	NR	29.9 [High]	31 [High]	11.3 [High]	30 [High]
Ukraine	NR	9.2 [High]	NR	1.3 [High]	NR
Southern Europe					
Albania	38.0 [Lower]	6.1 [Lower]	83 [Lower]	2.0 [Lower]	59 [Lower]
Bosnia and Herzegovina	NR	NR	NR	NR	NR
Catalonia	59.2 [Lower]	16.3 [Lower]	30 [Lower]	14.1 [Lower]	70 [Lower]
Croatia	59.0 [Lower]	13.6 [Lower]	43 [Lower]	7.8 [Lower]	72 [Lower]
Greece	0.8 [Lower]	15.0 [Lower]	59 [Lower]	2.0 [Lower]	157 [Lower]
Italy	65.0 [Lower]	19.0 [Lower]	58 [High]	11.7 [High]	90 [Lower]
Kosovo	50.0 [Lower]	7.2 [Lower]	NR	1.7 [Lower]	NR
Malta	NR	NR	NR	NR	NR
Montenegro	NR	NR	NR	NR	NR
North Macedonia	NR	1.3 [Lower]	62 [Lower]	0.3 [Lower]	NR
Portugal	NR	9.8 [High]	NR	13.1 [High]	NR
Serbia	NR	10.0 [Lower]	60 [Lower]	3.5 [Lower]	100 [Lower]
Spain	NR	8.2 [High]	NR	9.5 [High]	NR
Asia					
Armenia	NR	9.4 [Lower]	NR	7.9 [Lower]	NR
Azerbaijan	NR	NR	NR	NR	NR
Cyprus	NR	NR	NR	NR	NR
Georgia	NR	NR	NR	NR	NR
Israel	NR	10.3 [High]	NR	7.6 [High]	NR
Turkey	25.0 [Lower]	6.7 [Lower]	NR	8.5 [Lower]	NR

The classification of the data as either ‘high-quality data’ or ‘Lower quality data’ is presented in square brackets.

IVT – intravenous thrombolysis; EVT – endovascular therapy; NR – not reported

SUPPLEMENTARY FIGURES

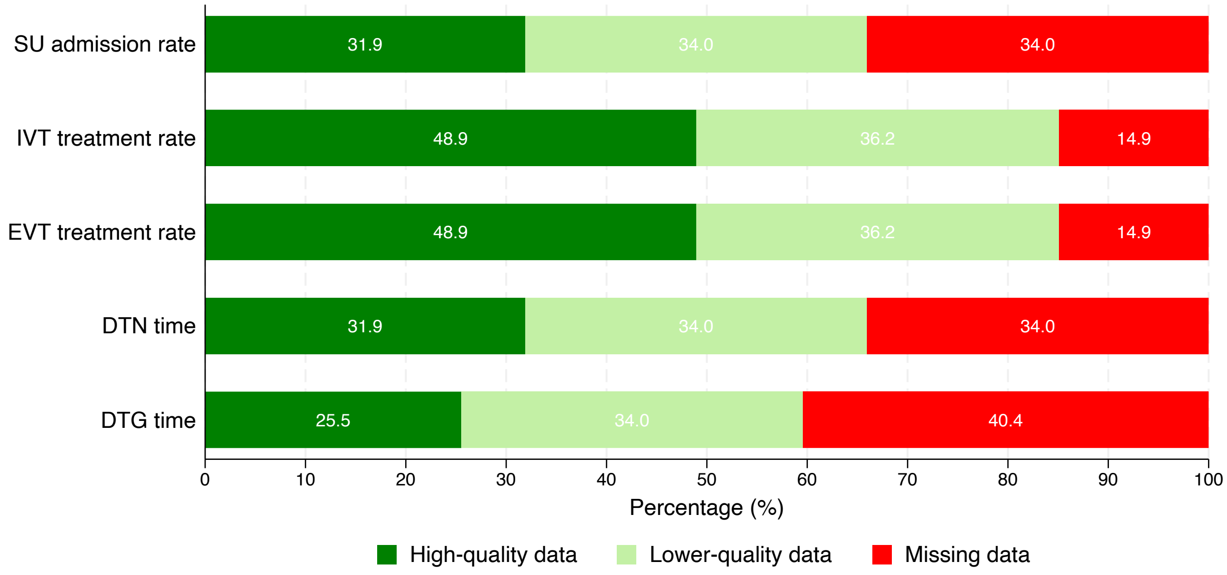

Supplementary figure 1*: Data quality distribution across the five key accessibility parameters. The figure displays the distribution of 'high-quality data', 'lower-quality data' and missing data for each parameter. SU – stroke unit; IVT – intravenous thrombolysis; EVT – endovascular treatment; DTN – door-to-needle; DTG – door-to-groin*

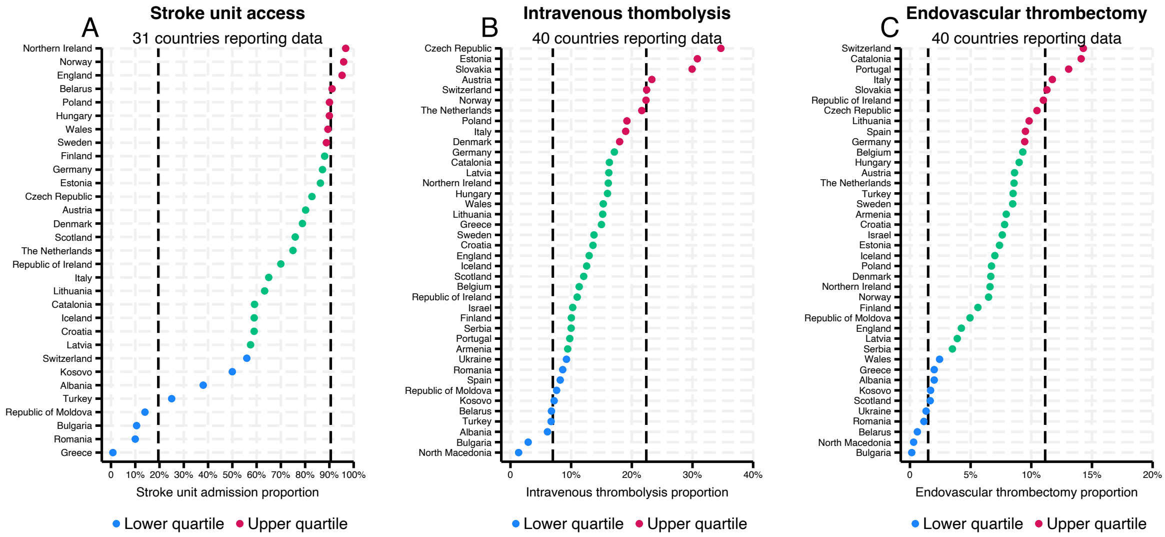

Supplementary figure 2: *Disparities in key accessibility parameters across countries. Panels represent the proportion of stroke unit admissions (A), intravenous thrombolysis (B), and endovascular thrombectomy (C). Each dot represents an individual country. The dashed lines indicate the median values for countries within the upper and lower quartiles quantifying the dept of the disparities.*

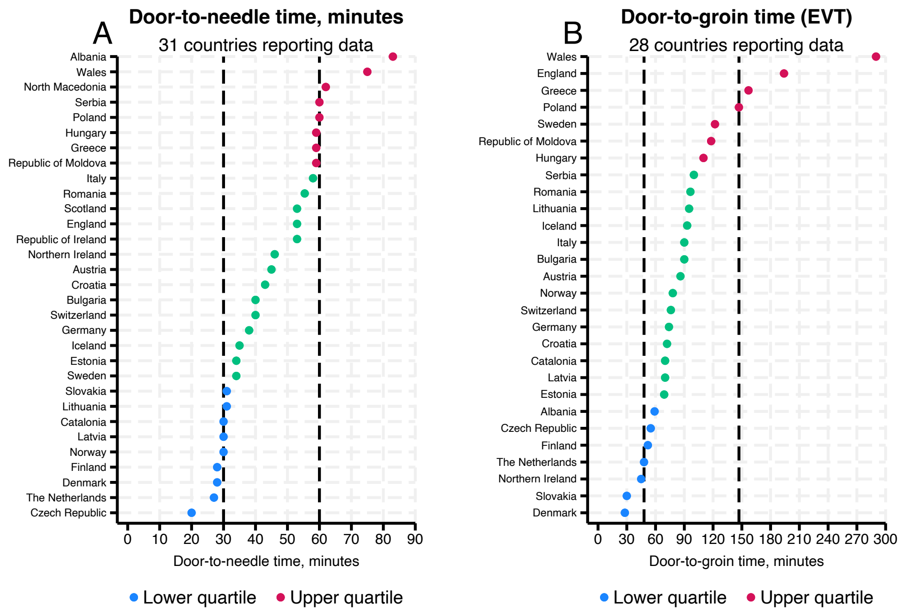

Supplementary figure 3: *Disparities in door-to-needle time and door-to-groin time between. Panels represent the median door-to-needle time (A), and door-to-groin time (B). Each dot represents an individual country. The dashed lines indicate the median values for countries within the upper and lower quartiles quantifying the dept of the disparities. IVT – intravenous thrombolysis; EVT – endovascular treatment*

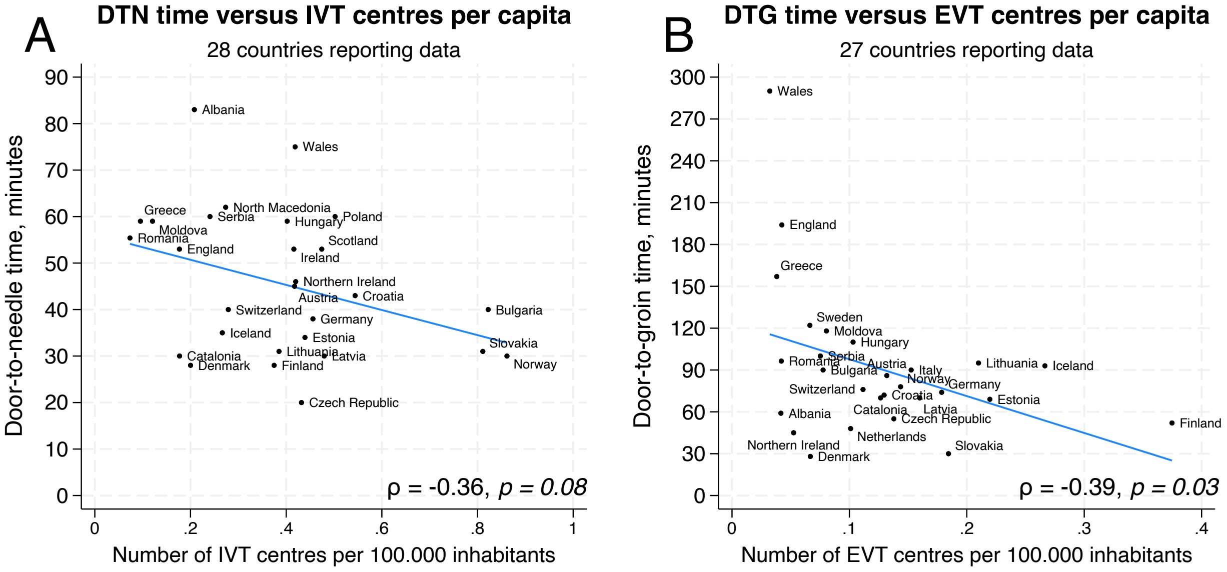

Supplementary figure 4: *Association between facility density and treatment delay. Panel A shows the relationship between the density of IVT-capable centers (per 100,000 inhabitants) and door-to-needle times (DTN) for intravenous thrombolysis. Panel B illustrates the association between the density of EVT-capable centers (per 100,000 inhabitants) and door-to-groin times (DTG) for endovascular treatment. IVT – intravenous thrombolysis; EVT – endovascular treatment*

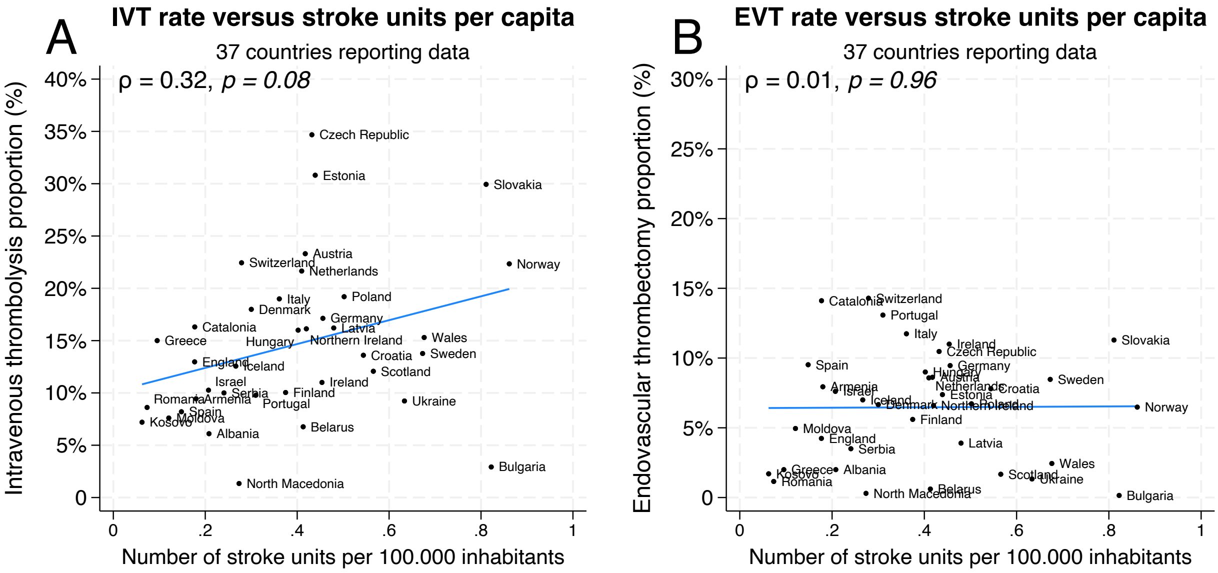

Supplementary figure 5: *Association between stroke unit density and accessibility of reperfusion therapy. Panel A show the association between the density of stroke units and the proportion of patients receiving IVT therapy. Panels B illustrate the association between the density of stroke units and the proportion of patients receiving endovascular therapy. IVT – intravenous thrombolysis; EVT – endovascular treatment*

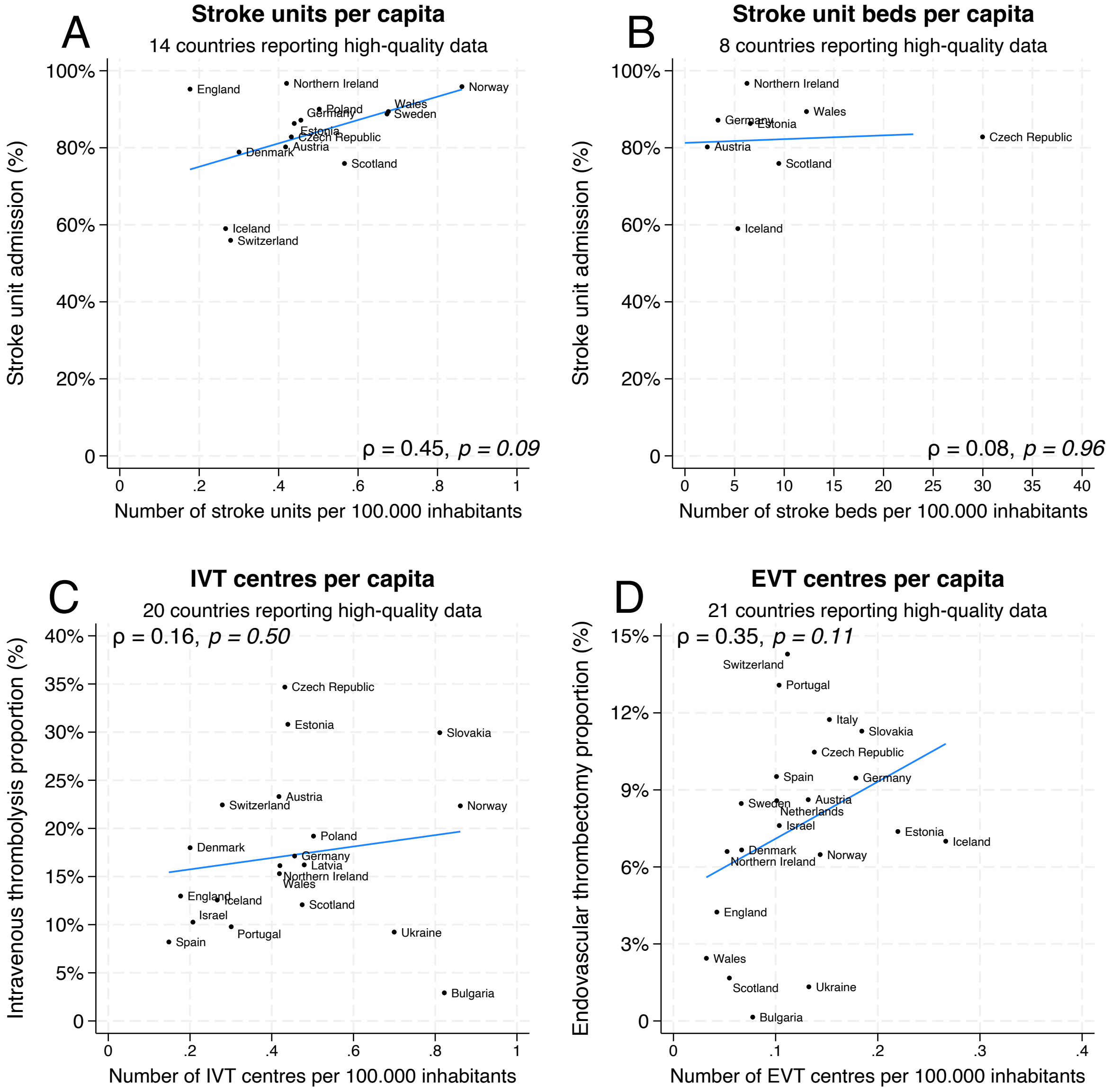

Supplementary figure 6: *Association between facility density and accessibility of stroke care in countries reporting high-quality data. Panel A and B show the association between the density of stroke units and stroke unit beds (no. per 100.000 population) and the proportion of stroke unit admissions. Panels C and D illustrate the association between the density of centers capable of providing reperfusion therapy (per 100,000 population) and the proportions of patients receiving such treatment. IVT – intravenous thrombolysis, EVT – endovascular therapy.*

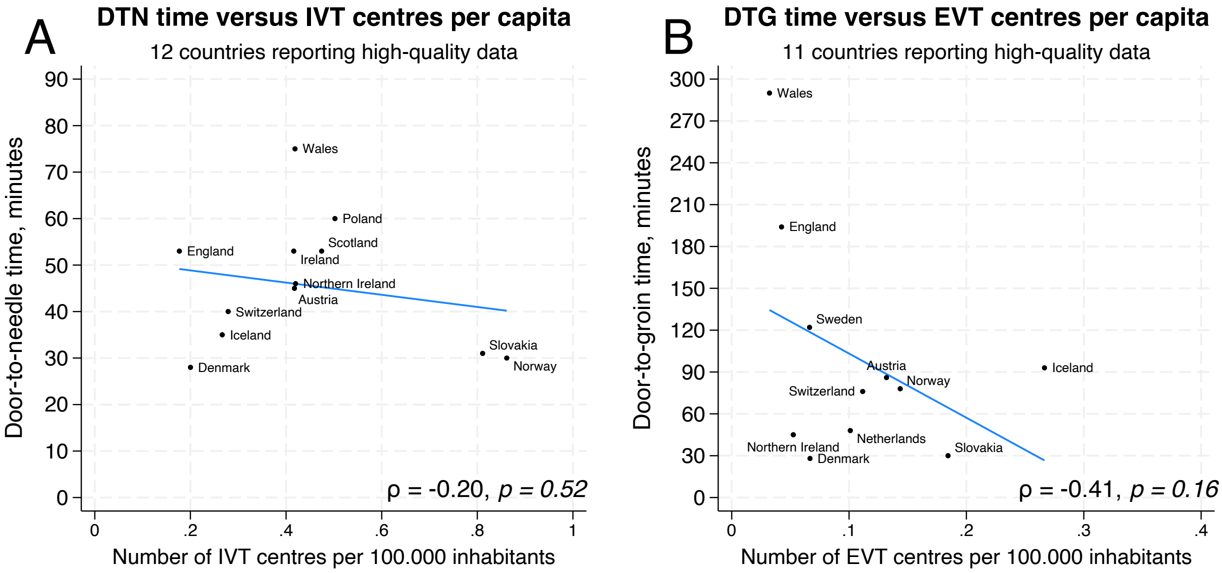

Supplementary figure 7: *Association between facility density and treatment delay in countries reporting high-quality data. Panel A shows the relationship between the density of IVT-capable centers (per 100,000 inhabitants) and door-to-needle times (DTN) for intravenous thrombolysis. Panel B illustrates the association between the density of EVT-capable centers (per 100,000 inhabitants) and door-to-groin times (DTG) for endovascular treatment. IVT – intravenous thrombolysis; EVT – endovascular treatment*

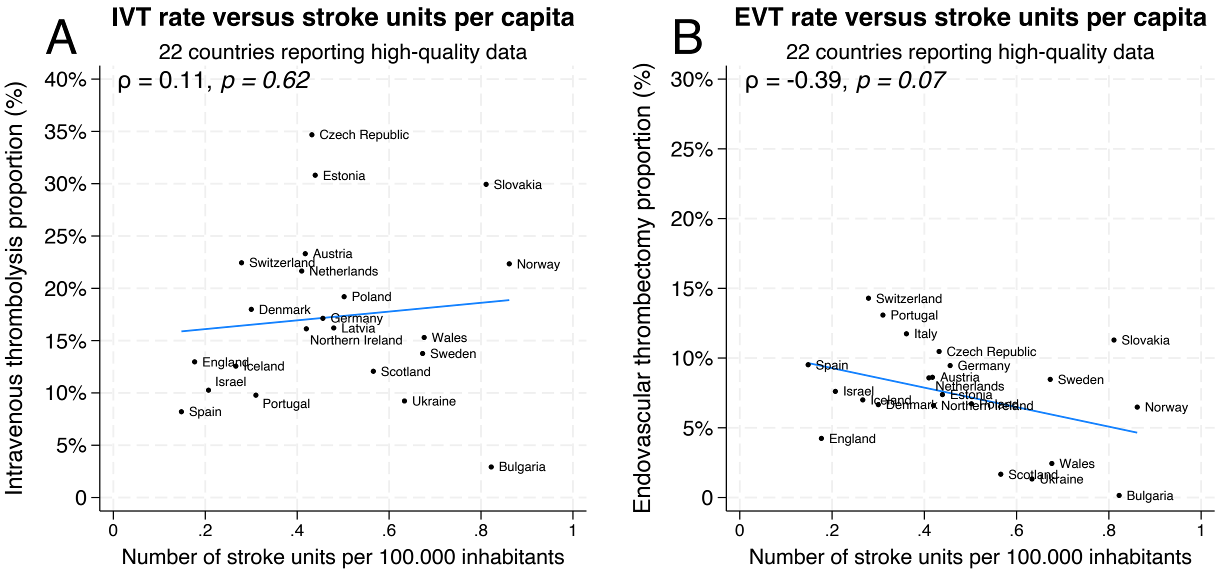

Supplementary figure 8: *Association between stroke unit density and accessibility of reperfusion therapy in countries reporting high-quality data. Panel A show the association between the density of stroke units and the proportion of patients receiving IVT therapy. Panels B illustrate the association between the density of stroke units and the proportion of patients receiving endovascular therapy. IVT – intravenous thrombolysis; EVT – endovascular treatment*

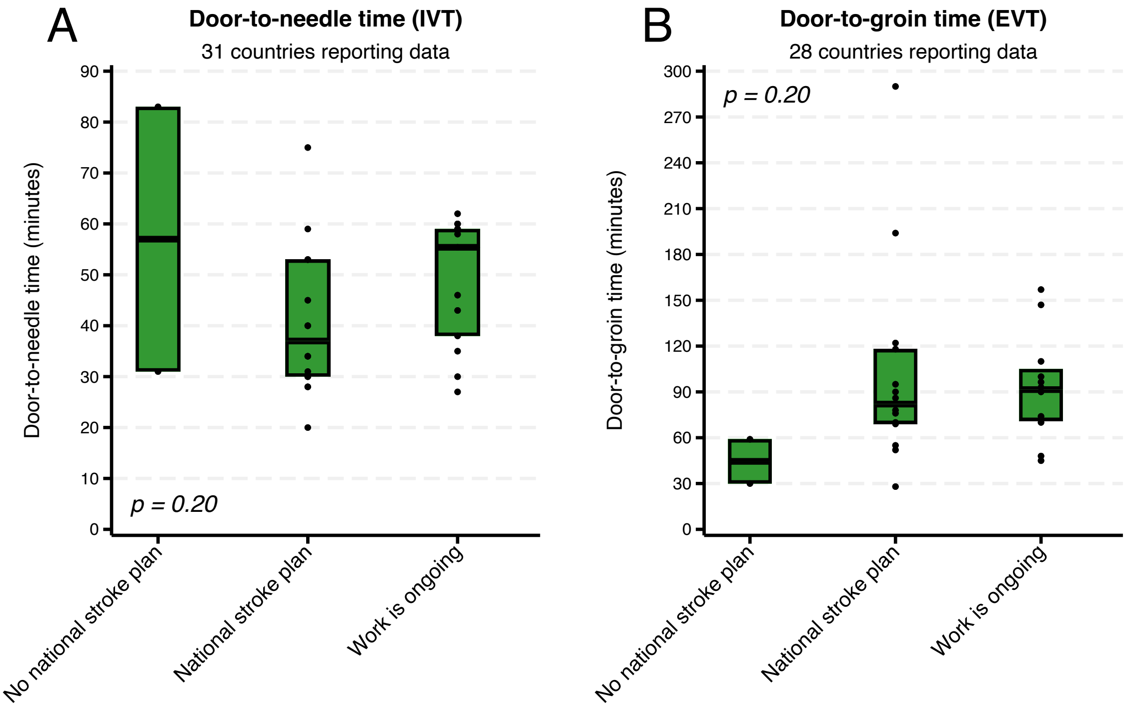

Supplementary figure 9: *Treatment delays stratified by national stroke plan implementation status. Bars represent the median (interquartile range) door-to-needle time (intravenous thrombolysis - panel A) and door-to-groin time (endovascular treatment - panel B). Individual country-level data points are superimposed as dots. IVT – intravenous thrombolysis; EVT – endovascular treatment*

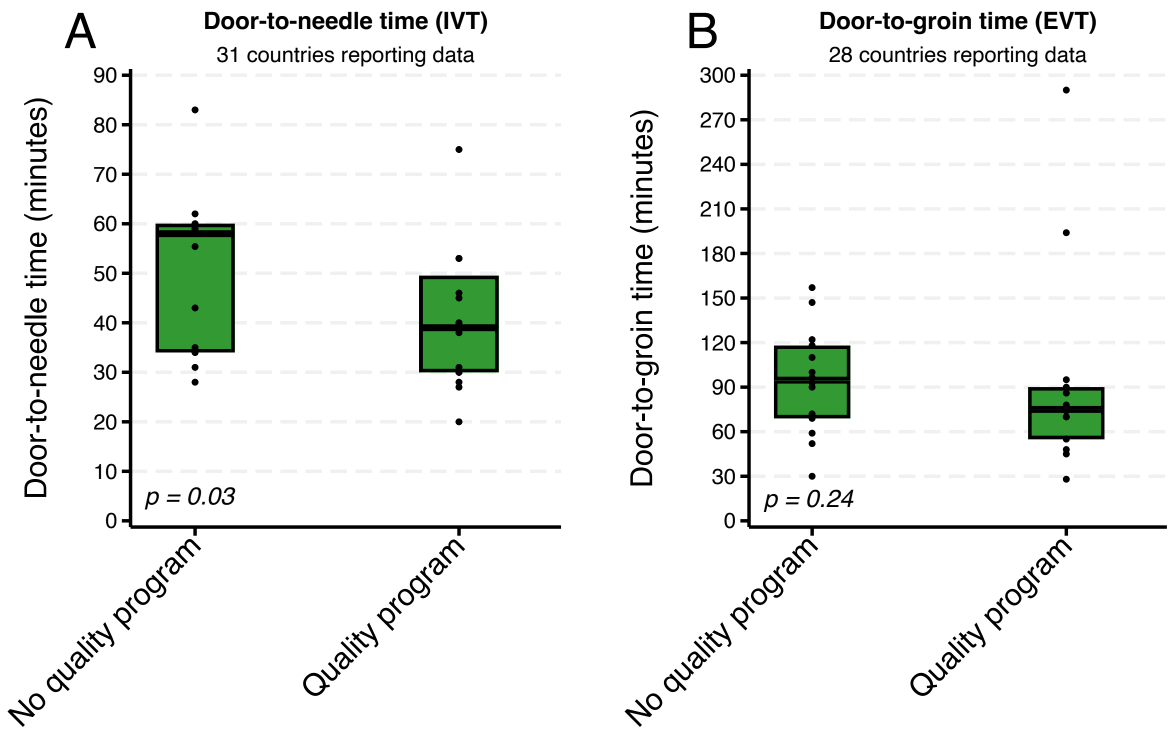

Supplementary figure 10: *Treatment delays stratified by the presence of a quality programme. Bars represent the median (interquartile range) door-to-needle time (intravenous thrombolysis - panel A) and door-to-groin time (endovascular treatment - panel B). Individual country-level data points are superimposed as dots. IVT – intravenous thrombolysis; EVT – endovascular treatment*

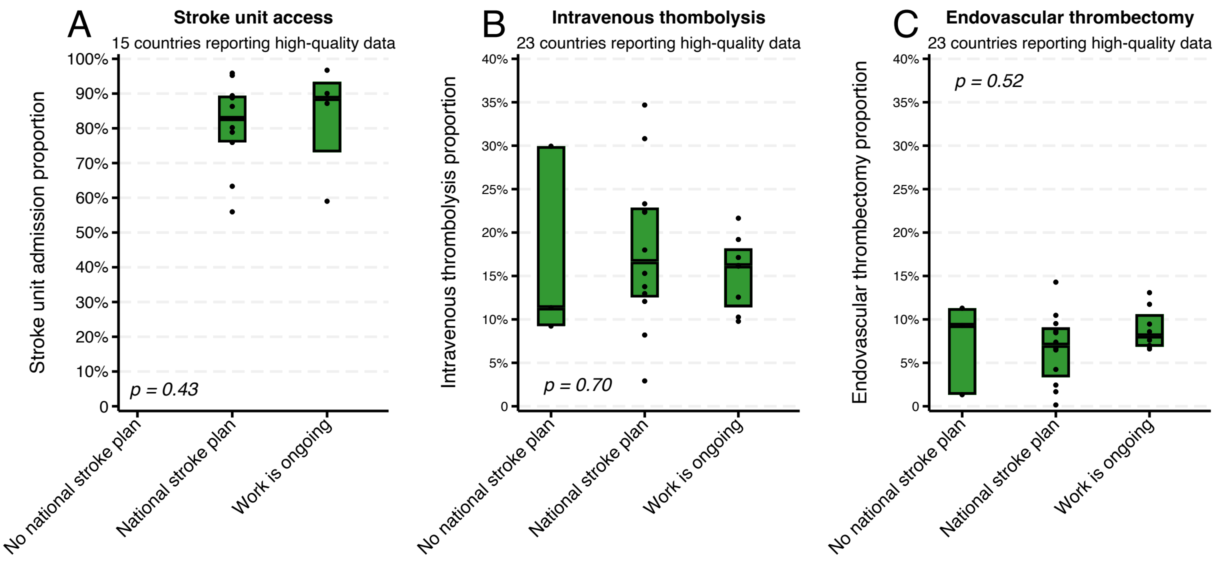

Supplementary figure 11: *Accessibility of stroke care stratified by national stroke plan implementation status in countries reporting high-quality data. Bars represent the median (interquartile range) proportions of stroke unit admissions (Panel A), intravenous thrombolysis (Panel B), and endovascular thrombectomy (Panel C). Individual country-level data points are superimposed as dots.*

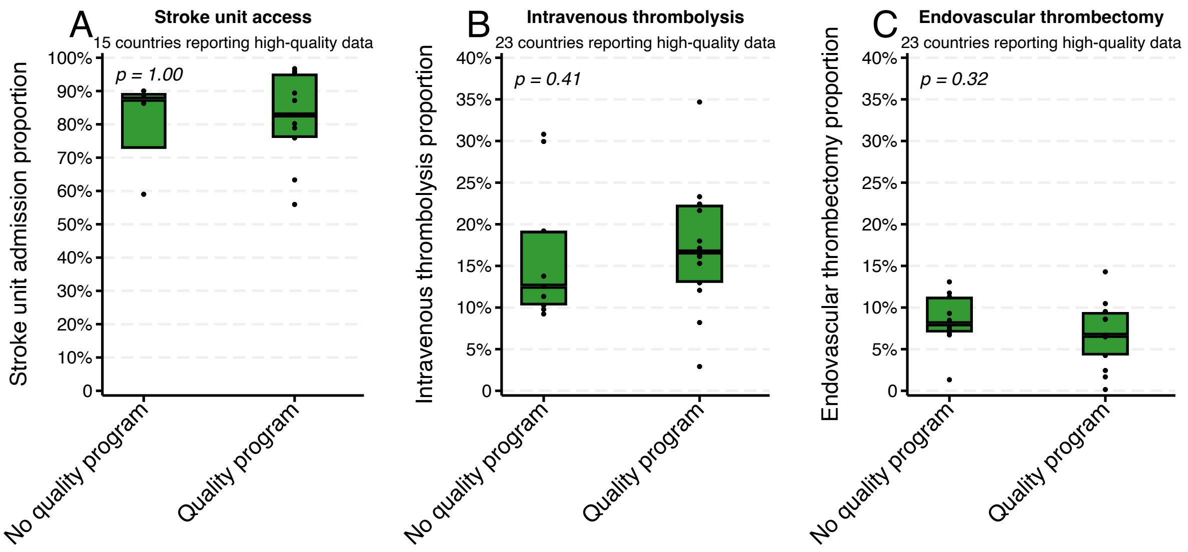

Supplementary figure 12: *Accessibility of stroke care stratified by the presence of a quality programme status in countries reporting high-quality data. Bars represent the median (interquartile range) proportions of stroke unit admissions (Panel A), intravenous thrombolysis (Panel B), and endovascular thrombectomy (Panel C). Individual country-level data points are superimposed as dots.*

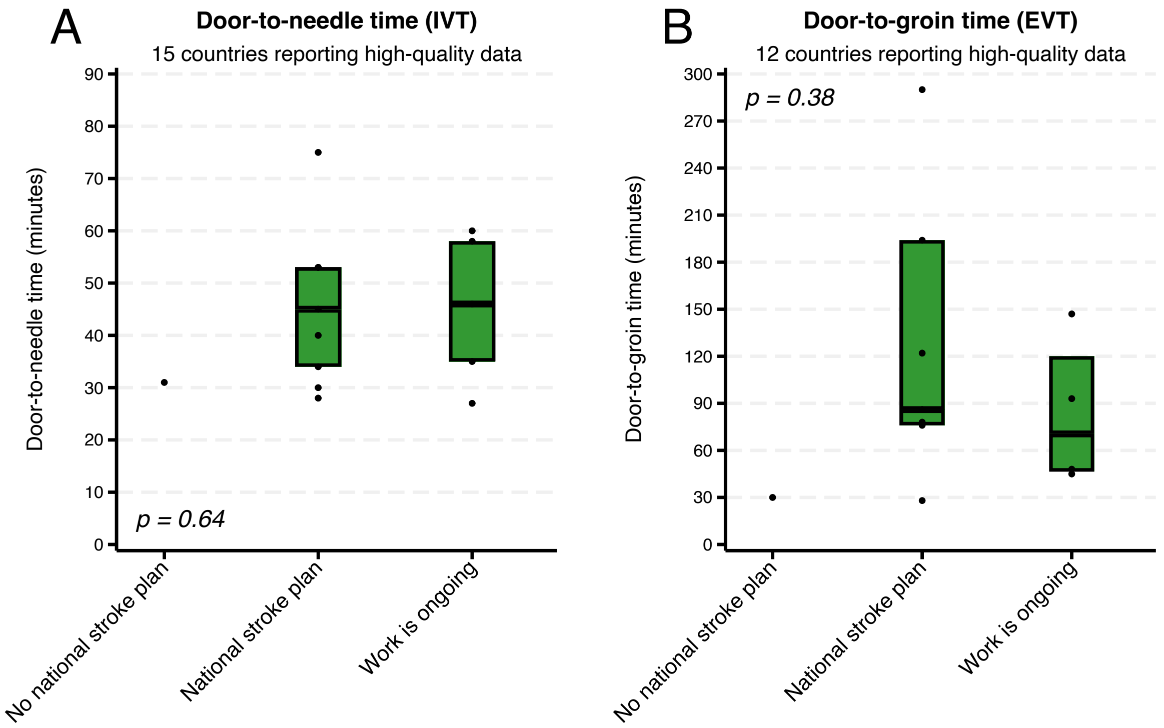

Supplementary figure 13: *Treatment delays stratified by national stroke plan implementation status in countries reporting high-quality data. Bars represent the median (interquartile range) door-to-needle time (intravenous thrombolysis - panel A) and door-to-groin time (endovascular treatment - panel B). Individual country-level data points are superimposed as dots. IVT – intravenous thrombolysis; EVT – endovascular treatment*

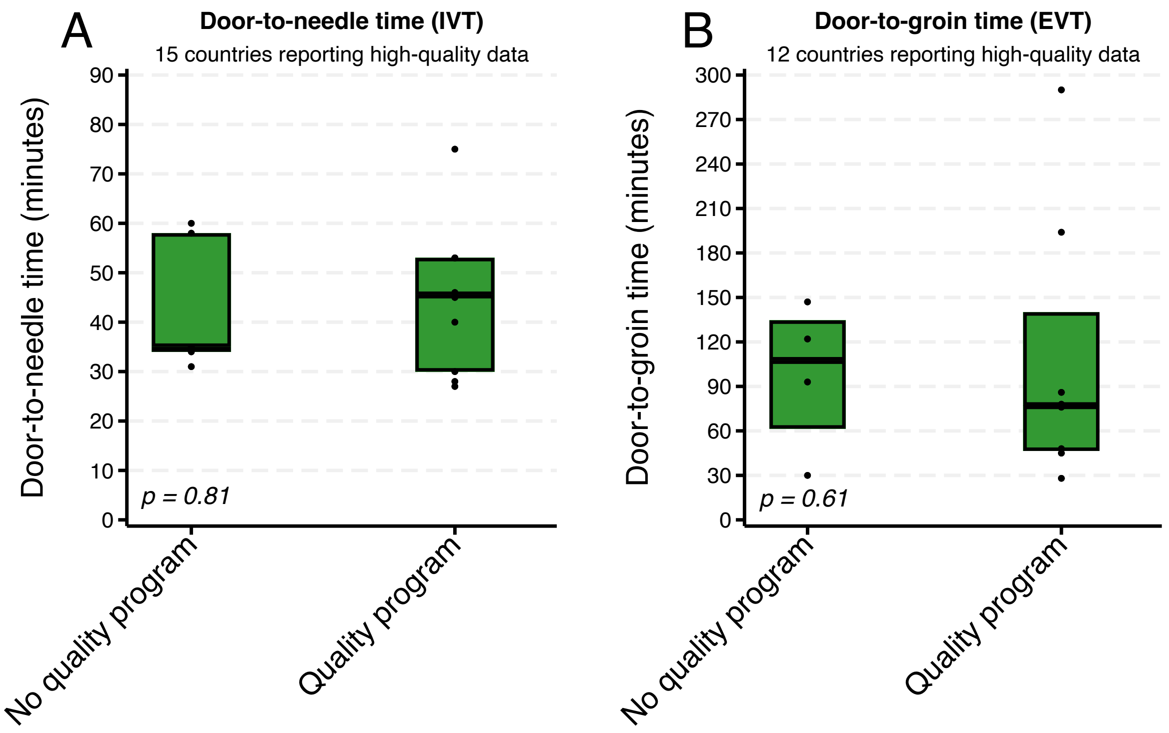

Supplementary figure 14: *Treatment delays stratified by the presence of a quality programme in countries reporting high-quality data. Bars represent the median (interquartile range) door-to-needle time (intravenous thrombolysis - panel A) and door-to-groin time (endovascular treatment - panel B). Individual country-level data points are superimposed as dots. IVT – intravenous thrombolysis; EVT – endovascular treatment*

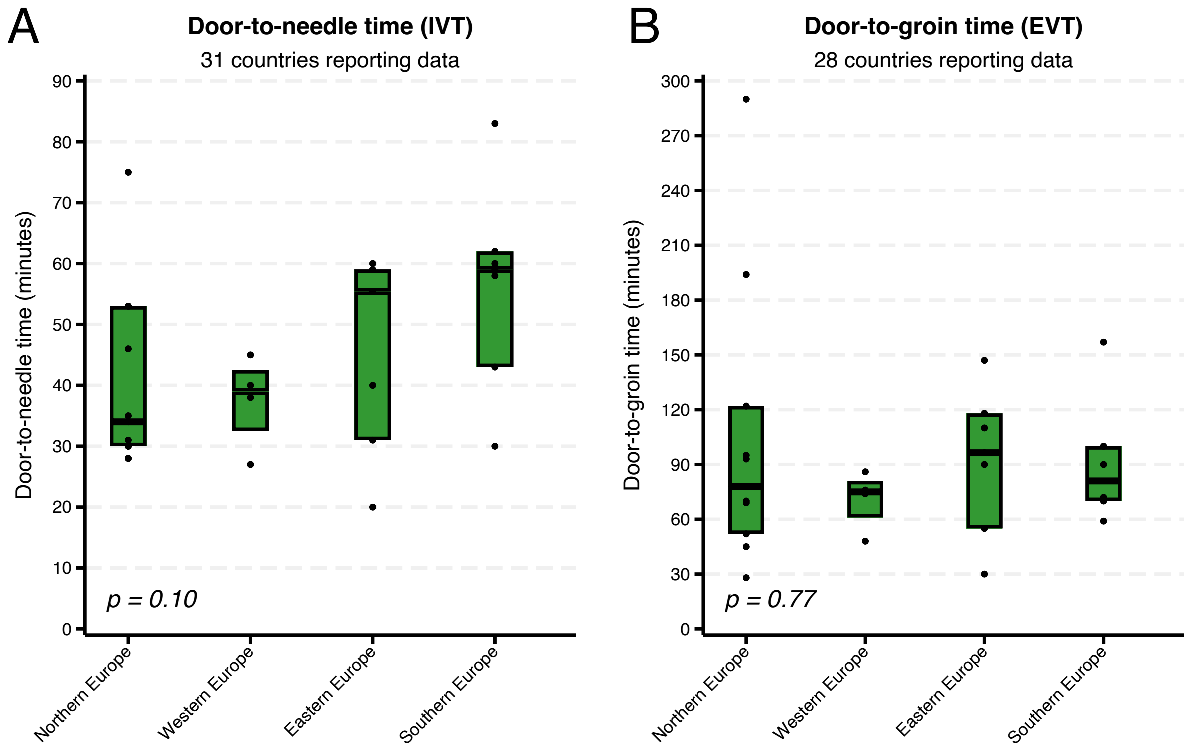

Supplementary figure 15: *Treatment delays stratified by geographic regions (UN geoscheme regions). Bars represent the median (interquartile range) door-to-needle time (intravenous thrombolysis - panel A) and door-to-groin time (endovascular treatment - panel B). Individual country-level data points are superimposed as dots. IVT – intravenous thrombolysis; EVT – endovascular treatment*

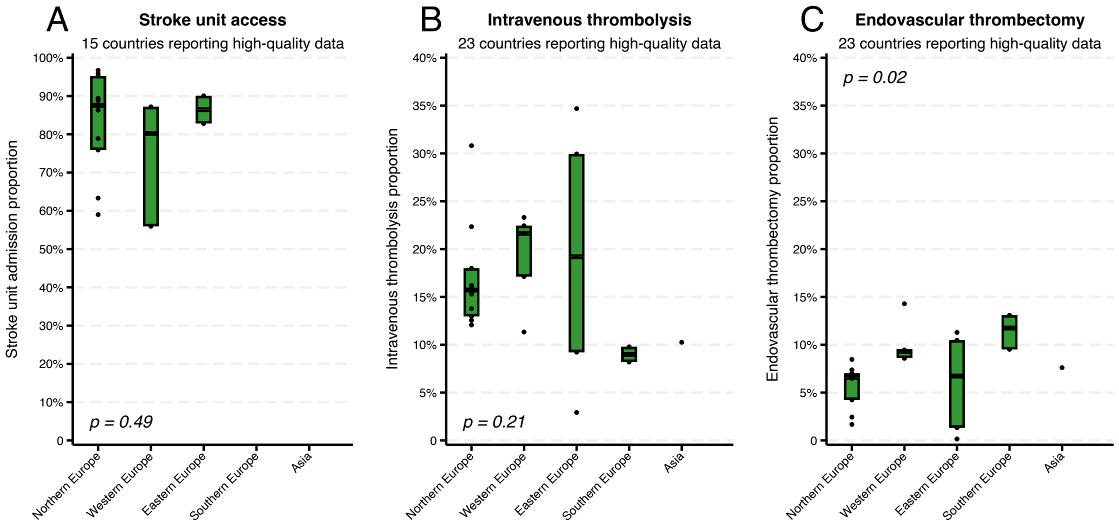

Supplementary figure 16: *Accessibility of stroke care stratified by geographic regions (UN geoscheme regions) in countries reporting high-quality data. Bars represent the median (interquartile range) proportions of stroke unit admissions (Panel A), intravenous thrombolysis (Panel B), and endovascular thrombectomy (Panel C). Individual country-level data points are superimposed as dots.*

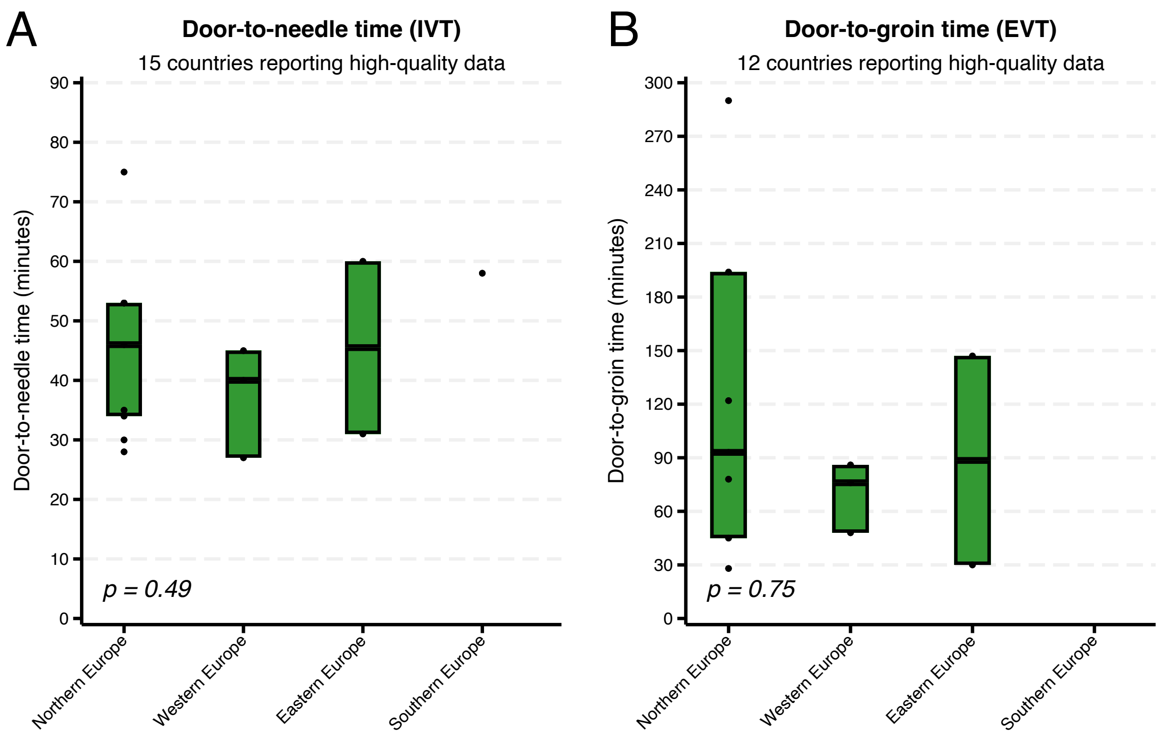

Supplementary figure 17: *Treatment delays stratified by geographic regions (UN geoscheme regions) in countries reporting high-quality data. Bars represent the median (interquartile range) door-to-needle time (intravenous thrombolysis - panel A) and door-to-groin time (endovascular treatment - panel B). Individual country-level data points are superimposed as dots. IVT – intravenous thrombolysis; EVT – endovascular treatment*
